# An LC-MS/MS method for protein detection based on a mass barcode and dual-target recognition strategy†

**DOI:** 10.1039/d0ra01783c

**Published:** 2020-04-23

**Authors:** Duo Li, Qinxin Song, Tengfei Li, Chang Shu, Shunli Ji, Chang Su, Yuwen Su, Li Ding

**Affiliations:** Key Laboratory of Drug Quality Control and Pharmacovigilance, Ministry of Education, School of Pharmacy, China Pharmaceutical University No. 24, Tongjiaxiang Nanjing 210009 China dingli@cpu.edu.cn; Department of Pharmaceutical Analysis, China Pharmaceutical University No. 639, Longmian Road Nanjing 210009 China; Department of Clinical Pharmacology, Sir Run Run Hospital, Nanjing Medical University Nanjing 211166 China suyuwen@njmu.edu.cn; School of Pharmacy, Nanjing Medical University Nanjing 211166 China

## Abstract

A mass barcode mediated signal amplification strategy was developed and applied to the determination of protein. A new compound, *N*′-((2-aminopyridin-3-yl)methylene)-5-(1,2-dithiolan-3-yl)pentanehydrazide (TAPA), was synthesized from the linker and the signal barcode, and used as the bonding barcode. For the realization of signal transduction, TAPAs and the target catcher aptamers, were both modified on gold nanoparticles (AuNPs) to establish the relationship between TAPAs and the target. Owing to the fact that the amount of TAPAs was much greater than the target, the signal of the target was not only transduced to the signal of the mass barcodes, but also amplified greatly. Thrombin, an important biomarker for coagulation abnormality diseases, was selected as a model analyte. Two kinds of thrombin recognition aptamers, aptamer 29 (apt29) and aptamer 15 (apt15), were modified onto the magnetic beads (MBs) and AuNPs, respectively. The modified AuNPs were further functionalized with lots of TAPA and formed apt15–AuNPs–TAPA. MBs–apt29 and apt15–AuNPs–TAPA could both recognize the target thrombin and form the sandwich complex (MBs–apt29/thrombin/apt15–AuNPs–TAPA). After the complex was separated by an extra magnetic field, NaClO oxidant solution was added to release the signal barcodes, 2-Amino-3-pyridinecarboxaldehyde (APA), which were then collected after centrifuging and analyzed by LC-MS/MS. Under optimized conditions, the mass response intensity was proportional to thrombin concentration in the range of 0.05–10 nM, with a 0.007 nM detection limit. This method was applied to the determination of thrombin in spiked serum samples, and the average recoveries ranged from 89.6% to 110.4%, which confirmed the applicability of this method.

## Abstract 2

A mass barcode-mediated signal amplification method was developed for the determination of protein by LC-MS/MS.

## Graphical abstract

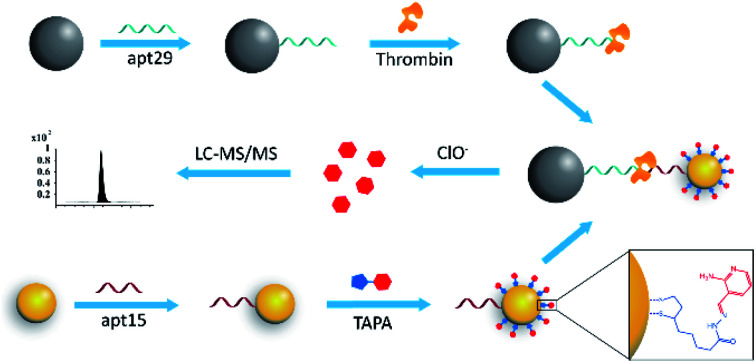

## 1. Introduction

Protein biomarkers, as novel indicators of biological states and pathological conditions, play vital roles in clinical diagnosis, therapy and prognosis.^1–3^ But due to the low abundance of some protein biomarkers, the dominant method currently used cannot meet the requirement of sensitivity in clinics.^4^ Thus, it is desirable to establish a sensitive and selective method for protein detection.^5–7^ To date, various methods have been reported to detect proteins based on fluorescence,^8–10^ colorimetry,^11^ electrochemical voltammetry,^12^ surface-enhanced Raman spectroscopy (SERS),^13^ and surface plasmon resonance (SPR).^14^ Liquid chromatography coupled with tandem mass spectrometry (LC-MS/MS), as a sensitive and accurate analytical instrument with absolute superiority in the field of small molecule detection, has the problem of poor ionization efficiency for proteins, owing to the high-molecular-weight. Chemical derivatization of targeted compounds can improve the detection performance of small molecules effectively,^15–18^ but for macromolecules such as protein, indirect measurement through mass barcode-mediated signal transduction strategy is a more appropriate way to solve this problem. Mass barcodes are kinds of small tags with strong mass response intensity, and could be modified on nanoparticles in large amounts together with targets to transduce and amplify the signal of targets. Now, some mass barcodes mediated methods have been reported for the detection of DNA^19,20^ and proteins.^21^ But most of those methods used matrix-assisted laser desorption ionization-time of flight (MALDI-TOF) mass spectrometry as the detectors, the more suitable mass method for quantitative analysis, LC-MS/MS method is still scarce.

In addition to the modification of the mass barcodes on nanoparticles, a catcher should be grafted to capture the target. Aptamers are a type of artificial oligonucleotides (ssDNA or RNA) selected by SELEX (systematic evolution of ligands by exponential enrichment).^22–24^ Compared with antibodies, aptamers as recognition elements, own numerous unique features, such as a wide range of targets, low cost, easy synthesis, high stability and convenient modification and immobilization. A variety of aptamer-based methods, combined with nanomaterials, have been reported in the detection of small organic molecules,^25^ proteins,^26–28^ viruses^29^ and tumor cells.^30^

In the process of the detection technology development, the significance of the advancement of nanomaterials is undeniable. Magnetic materials possess advantages including good biological compatibility, high specific surface area and easy to separation by an external magnetic field, and now, magnetic materials have been widely used as separation and enrichment carriers in complex sample analysis.^31–33^ Gold nanoparticles (AuNPs) occupy an important status in the construction of sensors in the past two decades. Their distinct properties, such as size-dependent colors, catalytic ability, good biocompatibility and low toxicity, make them outstanding probes in diverse sensing strategies.^34,35^

**Fig. 1:**
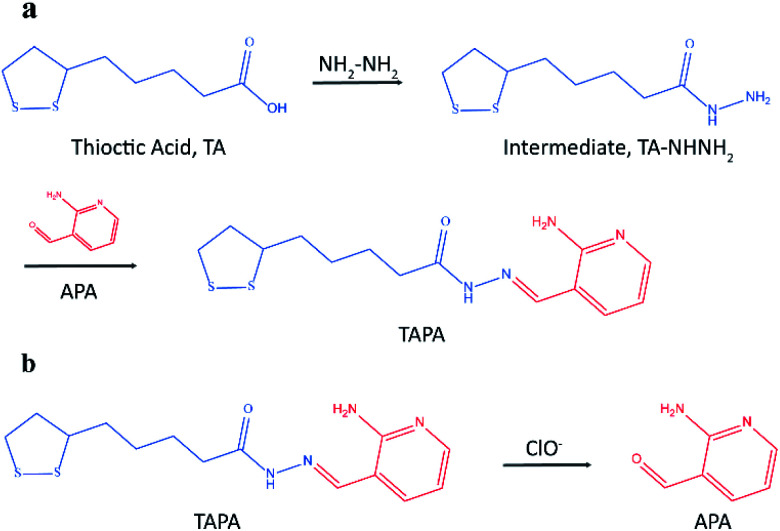
(a) The synthesic process of bonding barcode TAPA; (b) the generation process of signal barcode APA.

Herein, a novel LC-MS/MS method was presented based on mass barcode and dual-target recognition for the determination of thrombin (a model analyte). The design of mass barcode is a vital part of this method. Considering the characteristics of the carrier AuNPs and the requirements for the signal barcodes, the concept of linker was introduced in the design of mass barcode. As shown in Fig. 1a, the bonding barcode TAPA was synthesized from the linker (the intermediate, TA-NHNH_2_) and the signal barcode APA. This special compound TAPA could be modified on AuNPs *via* Au–S bond, and oxidized by ClO^−^ to produce the signal barcode APA (Fig. 1b).^36,37^ With the assistance of mass barcodes and sandwiched structure, a sensitive LC-MS/MS method for thrombin determination was successfully established. Under optimized conditions, this method exhibited good stability, high selectivity and excellent sensitivity with a detection limit of 0.007 nM. This method has potential application for thrombin detection in practice.

## 2. Experimental

### 2.1 Materials and reagents

Hydrogen tetrachloroaurate(iii) trihydrate (HAuCl_4_·3H_2_O), thrombin and aptamers were purchased from Shanghai Sangon Biotechnology Co., Ltd. (Shanghai, China). Methanol (HPLC grade) was purchased from Merck (Billerica, MA, USA). Nitrofurantoin (the internal standard, IS) was provided by Nine-Dinn Chemistry Co., Ltd. (Shanghai, China). Bovine serum albumin (BSA), Heparin and β-glucuronidase were supplied by Sigma-Aldrich (St. Louis., MO, USA). SYBR Green II was obtained from Beijing Solarbio Science & Technology Co., Ltd. (Beijing, China). Protein-free blocking buffer was purchased from Thermo Fisher Scientific (Madison, USA). The purified water was prepared by Direct-Q water purification system (Millipore, USA). The amino-modified magnetic beads (MBs, diameter 1–2 μm) were purchased from Baseline Chromatographic Technique Centre (Tianjin, China). The sequences of aptamers as follows,

Aptamer 29: 5′-NH_2_-T_12_-AGT CCG TGG TAG GGC AGG TTG GGG TGA CT-3′;

Aptamer 15: 5′-SH-T_5_-GGT TGG TGT GGT TGG-3′.

### 2.2 Apparatus

The microscopic image was recorded by a Hitachi HT7700 transmission electron microscopy (TEM) (Hitachi, Japan). The fluorescence spectra and UV-vis absorption spectra were acquired on a spectramax M2e multi-mode microplate reader (Molecular Devices, San Jose, CA, USA). The mass barcodes were analyzed by an Agilent 1260 Series liquid chromatograph (Agilent Technologies, Palo Alto, USA) equipped with an API 4000 triple quadrupole mass spectrometer (AB Sciex, Foster, USA).

### 2.3 Preparation of MBs with aptamer 29

The amino-modified MBs were conjugated with amine-modified aptamer 29 (apt29) using the glutaraldehyde cross-linking method.^38^ First, 1% of amine-modified MBs were washed three times and redispersed in PB buffer (10 mM, pH 8.0). Glutaraldehyde aqueous solution (50%) was added to obtain a final concentration of 2.5% and the mixture was shaken gently for 2 h. After washed by ultrapure water with an extra magnetic field, the particles were further dispersed in 3 × SSC buffer containing amine-modified apt29 and shaken overnight. Sodium borohydride aqueous solution was added to oxidize C=N and stabilize the cross-linking. The apt29 functionalized MBs were washed thrice with 3 × SSC buffer and resuspended in protein-free blocking buffer to minimize the nonspecific adsorption. Finally, the MBs–apt29 were redispersed in 25 mM Tris–HCl buffer (142 mM NaCl, 5 mM MgCl_2_, 15 mM KCl, pH 7.4) and stored at 4 °C before use.

### 2.4 Synthesis and functionalization of AuNPs

Citrate-stabilized AuNPs with an average diameter of 13 nm were synthesized according to the previous study.^39^ Briefly, aqueous HAuCl_4_ (100 mL; 1 mM) solution was heated to reflux in an oil bath under vigorous stirring, and then 10 mL tri-sodium citrate solution (38.8 mM) was added. The solution was kept boiling for 20 min, and the color would be changed constantly in the first 5 min. Then the oil bath was removed and stirring was kept until the solution cooled to room temperature. Finally, the solution was filtered through a 0.45 μm membrane and stored at 4 °C before use.

For the modification of AuNPs with apt15, 100 μM apt15 and 10 mM TCEP were mixed and incubated at room temperature for 1 h to reduce the disulfide bond. Then AuNPs (2.5 nM) was added and shaken gently for 16 h. After the reaction, 1 M NaCl was added in 5 times to get a final concentration of 0.1 M, and then the solution was aged for 36 h. After the solution was centrifuged at 6000 g for 20 min, the supernatant was removed and the precipitation was redispersed in PB buffer (10 mM, pH 8.0). The resulting AuNPs–apt15 were stored at 4 °C before use.

The synthesis procedure of TAPA was shown in ESI.† For the modification of AuNPs–apt15 with TAPA, AuNPs–apt15 and 100 μM TAPA were mixed and reacted at room temperature for 30 min. After the solution was centrifuged at 6000 g for 20 min, the supernatant was removed and the final obtained apt15–AuNPs–TAPA were redispersed in 25 mM Tris–HCl buffer (142 mM NaCl, 5 mM MgCl_2_, 15 mM KCl, pH 7.4).

### 2.5 Procedure for the detection of thrombin

In a typical experiment, 200 μL of samples were mixed with 50 μL of MBs–apt29, and the mixture was incubated at 37 °C for 2 h to capture the thrombin completely. Then the captured thrombin and MBs–apt29 were separated by an external magnetic field and redispersed with 100 μL of apt15–AuNPs–TAPA solution. The mixture was incubated at 37 °C for 2 h to form the sandwich complex MBs–apt29/thrombin/apt15–AuNPs–TAPA. After magnetic separation, the sandwich structure was carefully rinsed with 25 mM Tris–HCl buffer (142 mM NaCl, 5 mM MgCl_2_, 15 mM KCl, pH 7.4) and resuspended in 50 μL of ultrapure water. Then, 2.5 μL of 1% NaClO solution was added and produce signal barcodes APA, and nitrofurantoin solution was added as the internal standard. The mixture was vortexed for 1 min and centrifuged at 12 000 g for 10 min, then 30 μL of supernatant was transferred into an autosampler vial for LC-MS/MS analysis.

### 2.6 LC-MS/MS conditions

The chromatographic separation was achieved on a Hedera ODS-2 column (5 μm, 150 × 2.1 mm, Hanbon Science and Technology, China), protected by a security guard C18 column (5 μm, 4 × 2.0 mm, Phenomenex, Torrance, CA, USA). The mobile phase was composed of methanol (mobile phase A) and 0.1% formic acid solution (mobile phase B). The mass spectrometer was operated in the positive ESI† mode. Quantification was performed using multiple reaction monitoring of the transitions of *m*/*z* 123.1–78.0 for APA and *m*/*z* 239.0–122.0 for the IS nitrofurantoin. The system control and data analysis were carried out by Analyst (AB Sciex, version 1.5.2). The detail parameters were shown in ESI.† The product ion and multiple reaction monitoring mass spectra were shown in Fig. S2 and S3.†

## 3. Results and discussion

### 3.1 The principle of thrombin detection

Mass barcode mediated signal transduction method is an effective way to transduce the signal of protein for LC-MS/MS detection with the assistance of sandwich structure. In the process of method development, the design of mass barcode is an important part. An ideal mass barcode ought to satisfy the following points. First, the mass barcode could be modified on the carrier stably; second, the mass barcode could be dissociated from the carrier easily for subsequent LC-MS/MS detection; third, the signal barcode should possess a high mass response. In this method, AuNPs was selected as the carrier of mass barcodes, and thiols were the first choice for the modification of AuNPs because of the stable Au–S bonds. But the stable Au–S bonds also brought about the problem that it was hard to cleave the intact thiols from AuNPs for subsequent LC-MS/MS detection. So, the concept of linker was introduced to the design of mass barcode. The design of linker comprised two key points: the connections with the carrier and the signal barcode, respectively. For the bonding between the linker and the carrier, the stable Au–S bond was applicable. And for the bonding between the linker and the signal barcode, a cleavable bond was required. In this method, hydrazone bond, which could be oxidized by NaClO solution to release the signal barcode, was selected to connect the linker and the signal barcode. As shown in Fig. 1a, the linker (the intermediate, TA-NHNH_2_) was synthesized from thioctic acid and hydrazine, and the signal barcode APA was further attached by hydrazone bond. Then the cleavable bonding barcode TAPA, which could be modified on AuNPs *via* Au–S bonds and oxidized by ClO^−^ to release the signal barcode APA, was synthesized successfully.

**Scheme 1:**
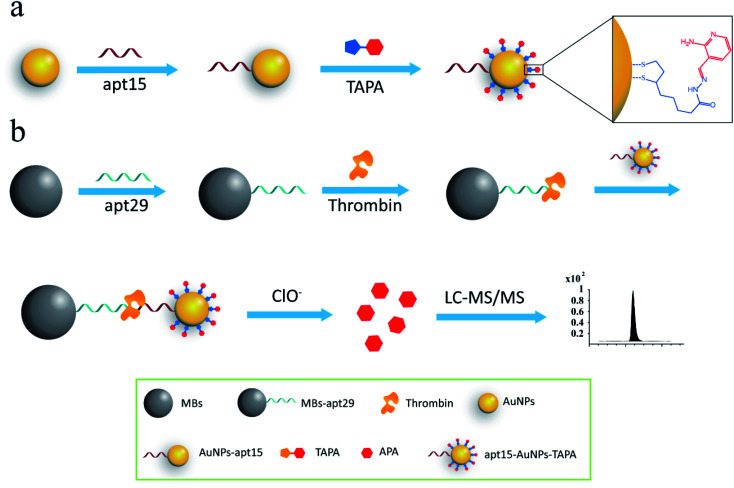
(a) The modification process of AuNPs with aptamers and mass barcodes; (b) schematic illustration of the assay for thrombin detection.

The process of the thrombin assay was illustrated in Scheme 1. Two kinds of thrombin recognition aptamers, apt29 and apt15 were employed for the specific recognition of thrombin and modified on MBs and AuNPs, respectively. The AuNPs–apt15 was further modified with a large amount of the bonding barcode TAPA and formed apt15–AuNPs–TAPA (Scheme 1a). As shown in Scheme 1b, thrombin was first captured by MBs–apt29 and then sandwiched by apt15–AuNPs–TAPA. Once the thrombin was captured by apt15–AuNPs–TAPA, the relationship between thrombin and mass barcodes were formed. The captured thrombin corresponded to the numerous mass barcodes on AuNPs. By this correspondence, the signal of thrombin was transduced to the signal of mass barcodes, and owing to the huge quantity difference, the signal was amplified greatly. Then, the sandwiched complex (MBs–apt29/thrombin/apt15–AuNPs–TAPA) was separated by an external magnetic field, and NaClO solution was added and reacted with TAPAs to produce the signal barcodes APAs, which were further collected through centrifugation and analyzed by LC-MS/MS.

### 3.2 Characterization

**Fig. 2:**
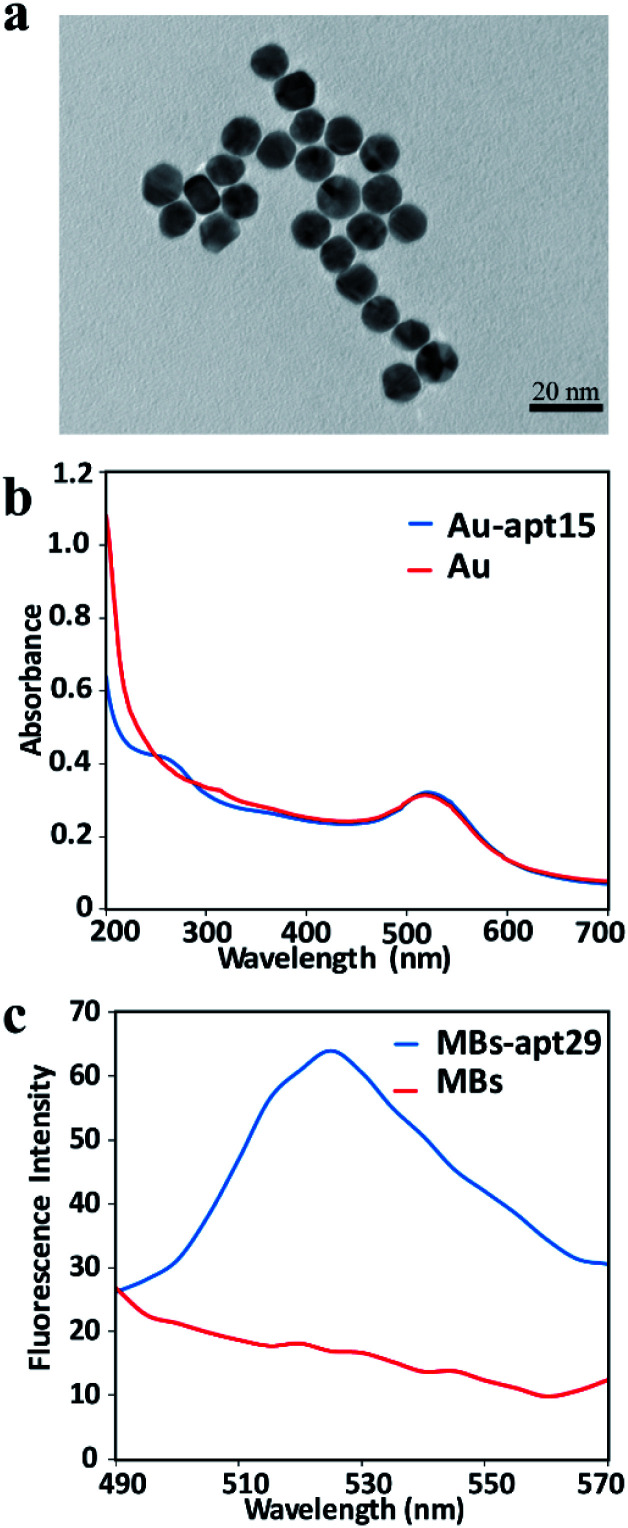
(a) The TEM image of AuNPs; (b) the UV-vis absorption spectrum of AuNPs and Au-apt; (c) the fluorescence spectra of MBs and MB-apt.

The as-prepared AuNPs were characterized by TEM to verify the size and morphology. The TEM image indicates that the AuNPs are spherical and uniformly dispersed, and the average diameter was about 13 nm (Fig. 2a). UV-vis spectra were used to confirm the modification of apt15 on AuNPs. Compared with the spectrum of AuNPs, the spectrum of AuNPs–apt15 showed an obvious absorbance around 260 nm (Fig. 2b), which was the effect of apt15, and this spectrum indicated the successful modification of apt15 on AuNPs. SYBR green II, a sensitive dye for detecting single-stranded DNA, was used to verify the modification of apt29 on MBs. As shown in Fig. 2c, MBs and MBs–apt29 were both mixed with SYBR green II, and an obvious peak from MBs–apt29 was showed at 525 nm, which indicated the successful preparation of MBs–apt29. It is worth to note that the fluorescence verification method with SYBR green II is more clear than UV-vis method, but the fluorescence could be quenched by AuNPs. So, the modification of apt15 on AuNPs was verified by UV-vis spectra, rather than SYBR green II.

### 3.3 Optimization of experimental conditions

**Fig. 3:**
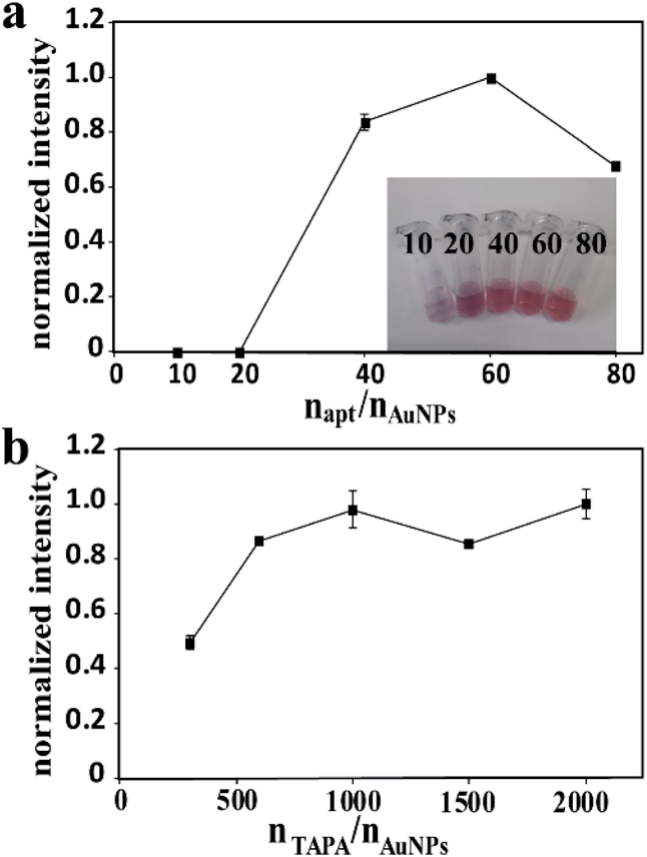
(a) The effect of different molar ratios of aptamers to AuNPs on the intensity, inset: Au-apts with different molar ratios of aptamers to AuNPs; (b) the effect of different molar ratios of TAPAs to AuNPs on the intensity.

For the probe apt15–AuNPs–TAPA, apt15 and TAPA were responsible for the thrombin recognition and the generation of signal barcodes, respectively. The proportional relationship between AuNPs, apt15 and TAPA made a great influence on the sensitivity of the method. To maximize the detection signal, the following experiments were designed. To optimize the molar ratio of apt15 to AuNPs, the ratio of TAPA to AuNPs was fixed, and different ratios of apt15 to AuNPs were tested. The highest signal intensity was obtained when the molar ratio of apt15 to AuNPs was 60 (Fig. 3a). Besides, if the proportion of aptamers was too low, the AuNPs–apt15 would agglomerate in the aging process. As the inset in Fig. 3a showed, the 10 times sample and the 20 times sample yielded color changes after the adding of NaCl solution, which implies the agglomeration of AuNPs–apt15. Similarly, for the optimization of the molar ratio of TAPA to AuNPs, the ratio of apt15 to AuNPs was fixed, and different ratios of TAPA to AuNPs were tested. As Fig. 3b showed, the signal did not increase when the amount of TAPA more than 1000 times to AuNPs. Finally, the ratio of AuNPs, apt15 and TAPA was determined to be 1 : 60 : 1000. Besides, the incubation conditions including pH, the concentration of NaCl and the incubation time were optimized. Details were given in the ESI (Fig. S4†). The following conditions were found to give best results: (a) the pH of incubation buffer was 7.4; (b) the concentration of NaCl in incubation buffer was 142 mM; (c) the incubation time was 2 h.

### 3.4 The stability of mass barcodes

Various researches have been reported about the utilization of hydrazone bonds in different types of drug delivery systems. The characteristics of being stable in mildly basic or neutral conditions and release under acidic conditions were the most concerned. However, the labile bonds are always troubled in the problem of stability. According to previous studies, the hydrazone bonds formed from aldehydes are more stable than the hydrazone bonds synthesized from ketones.^40^ Herein, an aldehyde compound, APA was chosen as the signal barcode, and the bonding barcode TAPA was proved to be stable for more than 72 h in buffer (Fig. S5†). Unlike the requirement of control release of drug delivery systems, the detection signal needs to be released in a short time. It is hard to meet the requirement of rapid release by self-hydrolysis. In pH 3 solution, the intensity of TAPA after 10 h was 60.3% of the original intensity (Fig. S6†), which means that TAPA was still 60.3% left after 10 h. The calculation equation was *R* = TAPA_10h_/TAPA_0_, where *R* was the intensity ratio, TAPA_10h_ was the TAPA intensity after 10 h in pH 3 solution and TAPA_0_ was the original TAPA intensity. So an oxidant NaClO was added to accelerate the process. This redox reaction was rapid. The signal of TAPA disappeared after the mix with NaClO solution for just 1 min, which proved that the reaction could be finished in 1 min. Besides, the strong oxidation of NaClO solution would raise concerns about the stability of APA. Three different concentrations of APA solution (0.1 μM, 1 μM, 10 μM) mixed with NaClO solution were tested. The final concentration of NaClO solution was 0.05%. After 12 h, the APA intensities of three concentrations were 98.6%, 104.1% and 102.0% of the original intensities respectively. The calculation equation was *R* = APA_12h_/APA_0_, where *R* was the intensity ratio, APA_12h_ was the APA intensity after 12 h and APA_0_ was the original APA intensity. The results confirmed that APA could be stable in 0.05% NaClO solution for at least 12 h.

### 3.5 Quantitative range and sensitivity

Under the optimized detection conditions, various concentrations of the thrombin standard solution were detected. A good linear relationship was obtained between the response intensity and thrombin concentration in the range of 0.05–10 nM. The regression equation was *y* = 0.9002*x* + 0.6597, where *y* is the peak-area ratio of APA to IS and *x* is thrombin concentration with a correlation coefficient of 0.9914 (Fig. S7†). The limit of detection was estimated to be 0.007 nM at a signal-to-noise ratio of 3. Compared with other methods reported recently for thrombin detection (Table 1), our method has a lower or comparable detection limit.

**Table 1:** Comparison of different methods reported recently for thrombin detection

Analytical methods	Linear range (nM)	Detection limit (nM)	Reference
Fluorescence	0.01–50	0.007	2
Colorimetry	0.5–10	0.88	6
Fluorescence	0.37–50	0.37	10
MALDI-TOF	2.78–278	2.36	24
LC-MS/MS	0.5–50	0.3	27
Electrochemical	1–10 000	0.35	32
LC-MS/MS	0.05–10	0.007	This work

### 3.6 Selectivity

**Fig. 4:**
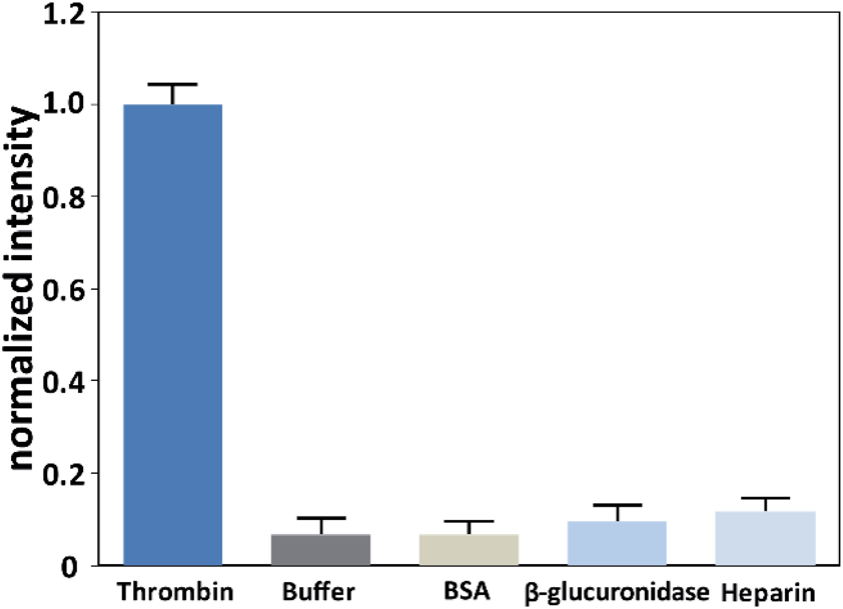
Responses of the assay to thrombin (0.1 nM), blank buffer sample, heparin (10 nM), BSA (10 nM) and β-glucuronidase (10 nM).

To investigate the selective of the assay for thrombin, blank buffer samples and several non-target proteins including BSA (10 nM), β-glucuronidase (10 nM), and heparin (10 nM) were evaluated. As shown in Fig. 4, the signal of thrombin with a concentration of 0.1 nM was much higher than the blank buffer samples and the non-target proteins with a 100-times higher concentration. This result confirmed that this method possessed excellent selectivity toward the target thrombin.

### 3.7 Analytical application in real sample

In order to confirm the feasibility and accuracy of the method for real sample analysis, the analytical method was applied to detect thrombin in spiked serum. Three 100-folds diluted serum samples containing different concentrations of thrombin (0.1, 1 and 10 nM) were detected. As shown in Table 2, the average recoveries were in a range of 89.6–110.4% with RSDs less than 10.2%, which were acceptable for quantitative analysis of biological samples.

**Table 2:** Determination of thrombin in spiked serum samples (*n* = 3)

Spiked (nM)	Found (nM)	Recovery (%)	RSD (%)
0.1	0.110	110.4	10.2
1.0	0.896	89.6	6.3
10.0	9.66	96.6	5.1

## 4. Conclusion

In summary, we developed a sensitive and selective method based on mass barcode for the detection of thrombin by LC-MS/MS without enzymolysis. TAPA, a hydrazone compound could be oxidized by NaClO solution to produce the signal barcode APA, was synthesized and used as the bonding barcode to transduce the signal of thrombin, and owing to the huge difference between the amount of mass barcode and thrombin, the signal was amplified greatly. The method displayed good sensitivity, excellent selectivity and favorable stability, indicating a potential application for thrombin detection in clinical diagnosis. Furthermore, the sensitivity could be further increased by using other aldehyde compounds with higher mass response as the signal barcodes. The concept of this method could be easily expanded to detect other proteins.

## Conflicts of interest

There are no conflicts of interest to declare.

## Supplementary Material

RA-010-D0RA01783C-s001
