# Supplementary material for: An LC-MS/MS method for protein detection based on a mass barcode and dual-target recognition strategy
Source: RSC Adv. 2020 Apr 23;10(27):16094–100. doi: 10.1039/d0ra01783c (PMC9052937; doi:10.1039/d0ra01783c)
Supplement: RA-010-D0RA01783C-s001 [file RA-010-D0RA01783C-s001.pdf]

Electronic Supplementary Material (ESI) for RSC Advances.
This journal is © The Royal Society of Chemistry 2020

An LC-MS/MS method for protein detection based on mass barcode and dual-target recognition strategy

Duo Li,[ab] Qinxin Song,[ab] Tengfei Li, [ab] Chang Shu, [ab] Shunli Ji, [ab] Chang Su,[c] Yuwen Su*[cd] and Li Ding*[ab]

[a] *Key Laboratory of Drug Quality Control and Pharmacovigilance, Ministry of Education, School of Pharmacy, China Pharmaceutical University, No.24, Tongjiaxiang, Nanjing 210009, China*

[b] *Department of Pharmaceutical Analysis, China Pharmaceutical University, No.639, Longmian Road, Nanjing 210009, China*

[c] *Department of Clinical Pharmacology, Sir Run Run Hospital, Nanjing Medical University, Nanjing 211166, China*

[d] *School of Pharmacy, Nanjing Medical University, Nanjing 211166, China*

E-mail address:
Yuwen Su: suyuwen@njmu.edu.cn
Li Ding：dingli@cpu.edu.cn;

1. Synthesis of TAPA

1.1 The synthesis of TA-COOEt.

1.03 g of TA, 0.31 g of DMAP, 4.24 mL of ethanol and 40 mL of CH_2Cl_2 were placed in a flask and stirred. The reaction mixture was cooled to 0 °C, and a solution of 1.54 g DCC in 10 mL CH_2Cl_2 was added dropwise. The reaction mixture was stirred at 0 °C for 1 h and then stirred at room temperature for 24 h. The precipitate was filtered and evaporated to give a yellow oil. The mixture was purified by flash chromatography and evaporated to give a yellow oil.

1.2 The synthesis of TA-$NHNH_2$.

1.0 g of TA-COOEt, 1.4 mL of $NH_2NH_2{\cdot}H_2O$, and 18 mL of methanol were mixed and stirred at 40 °C for 12 h. The mixture was diluted with ethyl acetate and the organic layer was washed with brine, dried with $MgSO_4$, then filtered and concentrated. The mixture was purified by flash chromatography and evaporated to give a yellow solid.

1.3 The synthesis of TAPA.

132 mg of TA-$NHNH_2$ and 97.7 mg of APA were dissolved in 30 mL of methanol. 50 μL of TFA was added and the mixture was stirred at room temperature for 24 h in the dark. The mixture was concentrated and purified by flash chromatography and evaporated to give a yellow solid. The mass spectrum of TAPA was shown in Fig. S1.

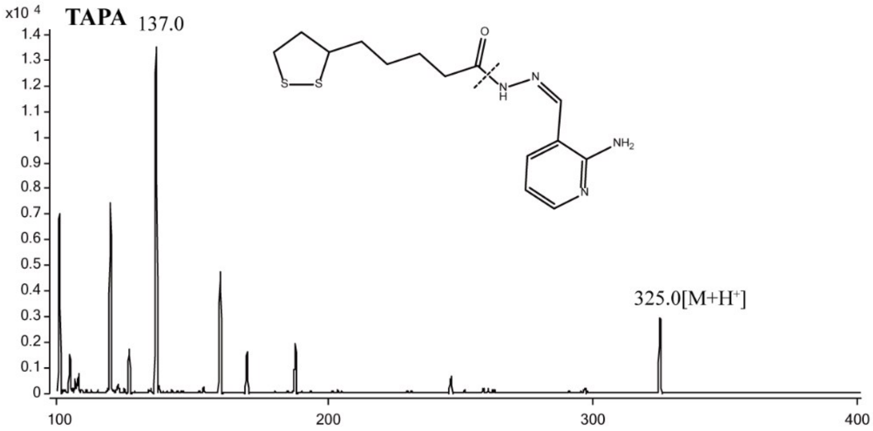

Fig. S1 Product ion mass spectrum of TAPA.

2. LC-MS/MS conditions

The chromatographic separation was achieved on a Hedera ODS-2 column (5 μm, 150 × 2.1 mm, Hanbon Science and Technology, China), protected by a security guard C_{18} column (5 μm, 4 × 2.0 mm, Phenomenex, Torrance, CA, USA). The mobile phase was composed of methanol (mobile phase A) and 0.1% formic acid solution (mobile phase B). The gradient elution produce was started at 60% A for 2 min, changing linearly to 100% A within 0.5 min, then maintained for 2 min and finally brought back to the initial condition during 0.5 min and re-equilibrated for 4.0 min. The injection volume was 5 μL. The autosampler was maintained at 8 °C.

The mass spectrometer was operated in the positive ESI mode. Quantification was performed using multiple reaction monitoring of the transitions of m/z 123.1 – 78.0 for APA and m/z 239.0 – 122.0 for the IS nitrofurantoin. Declustering Potential (DP) set for APA and IS was 60 V and 80 V, respectively. The Collision Energy (CE) was set at 32 V and 28 V for APA and IS, respectively. The collision gas and curtain gas were maintained at 12 and 30 psi, respectively. The ion source gas 1 and ion source gas 2 were both 30 psi. The ionspray voltage and temperature were kept at 4000 V and 450 °C. The system control and data analysis were carried out by Analyst (AB Sciex, version 1.5.2).

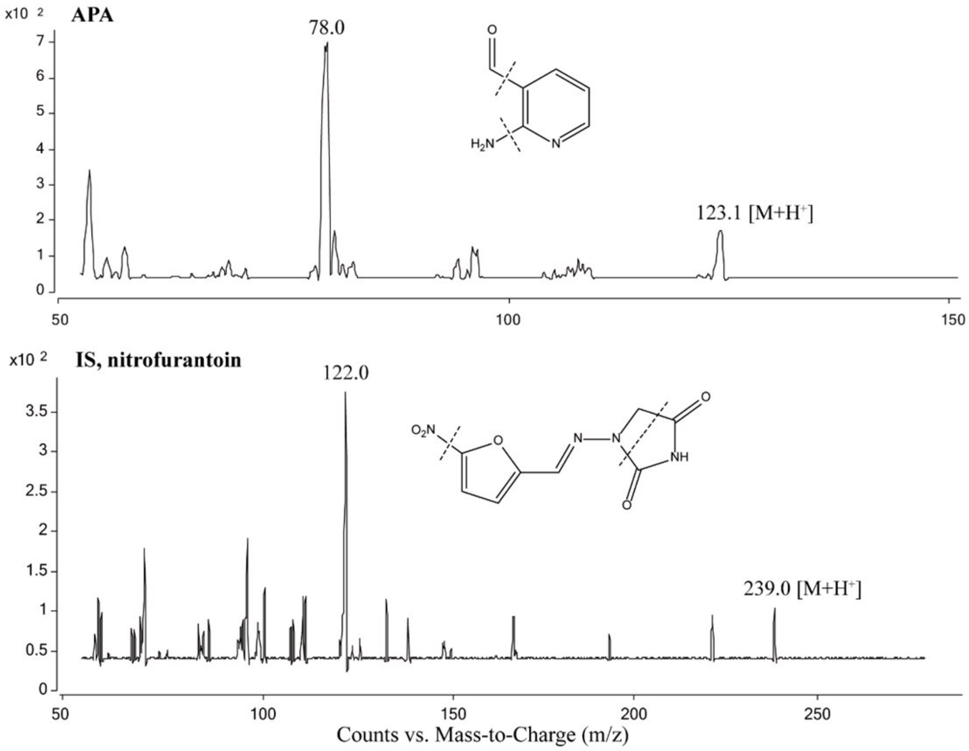

Fig. S2 Product ion mass spectra of APA and nitrofurantoin.

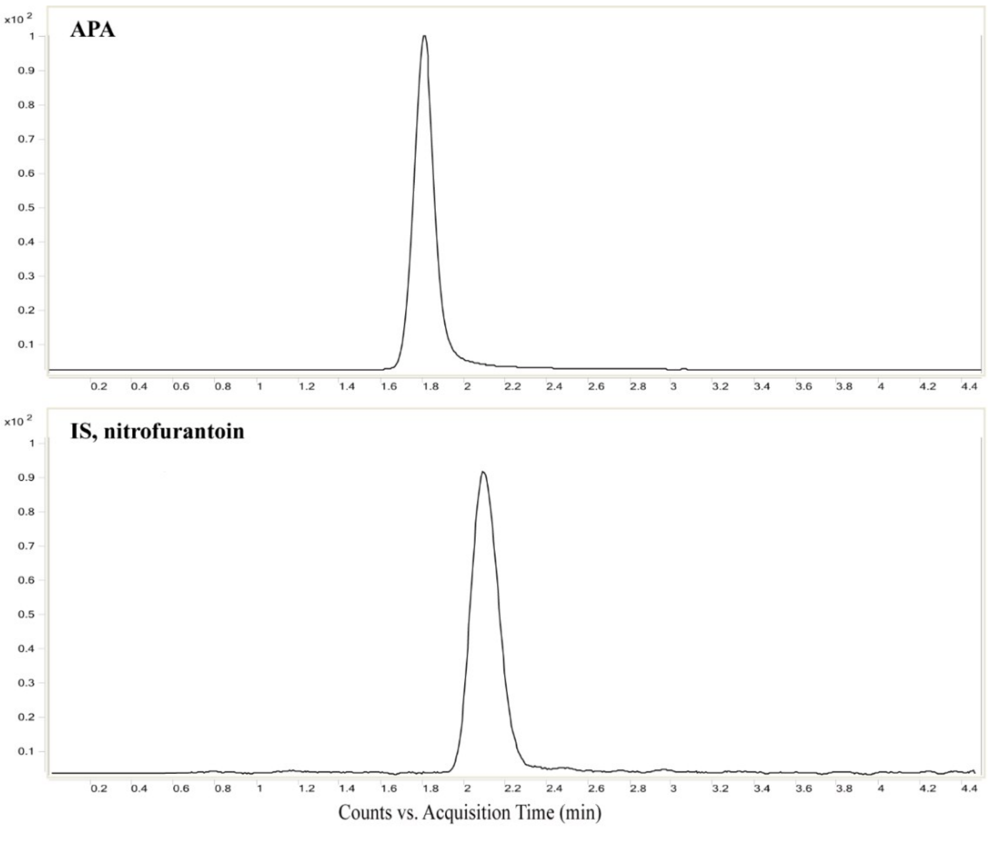

Fig. S3 Multiple reaction monitoring mass spectra of APA and nitrofurantoin.

3. Optimization of experimental conditions

In order to obtain the best performance, some experimental conditions such as pH, the concentration of NaCl and the incubation time were optimized.

To investigate the effect of pH on the connection between aptamers and thrombin, a series of 25 mM Tris-HCl buffer (142 mM NaCl, 5 mM $MgCl_2$, 15 mM KCl) with different pH (6.0, 6.5, 7.0, 7.4, 8.2, 9.0) was tested. As shown in Fig. S4a, there was no obvious change of mass intensities with the change of pH, so we chose the pH of physiological environment 7.4 as the final pH condition.

Aptamers need the assistance of metal ions to form G-quadruplex for the recognition of thrombin. In this experiment, different concentrations of NaCl (50, 100, 142, 200, 250 mM) in 25 mM Tris-HCl buffer was investigated. As shown in Fig. S4b, the intensity increased gradually with the increase of the concentration from 50 – 142 mM, and then the increase of concentration has little influence on the intensity. So 142 mM was regarded as the best concentration of NaCl in the system.

Incubation time is also important factor for the reaction. As shown in Fig. S4c, it can be seen that when aptamers reacted with thrombin for 2 h, the intensity reached a plateau and kept stable. Therefor, 2 h is consider as the optimum incubation time.

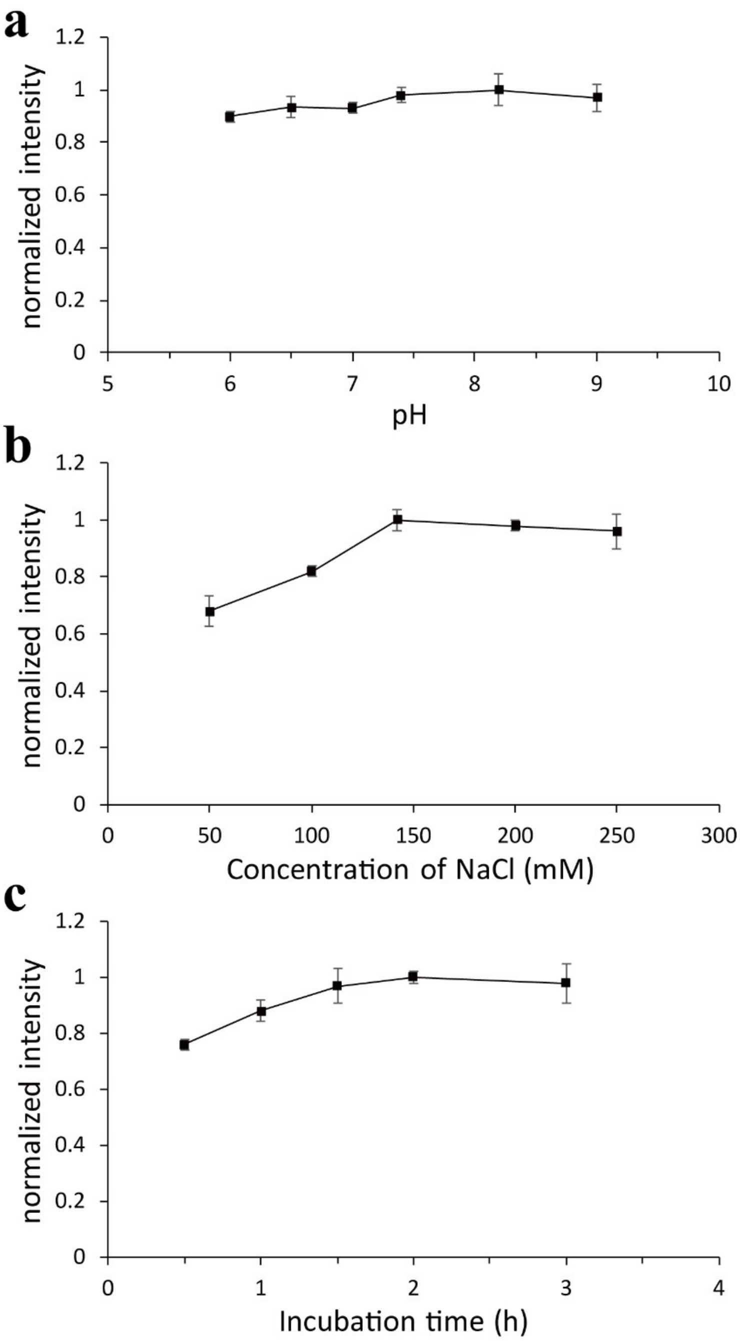

Fig. S4 The influence of different conditions on the mass response, (a) the effect of pH; (b) the effect of the concentration of NaCl; (c) the effect of incubation time.

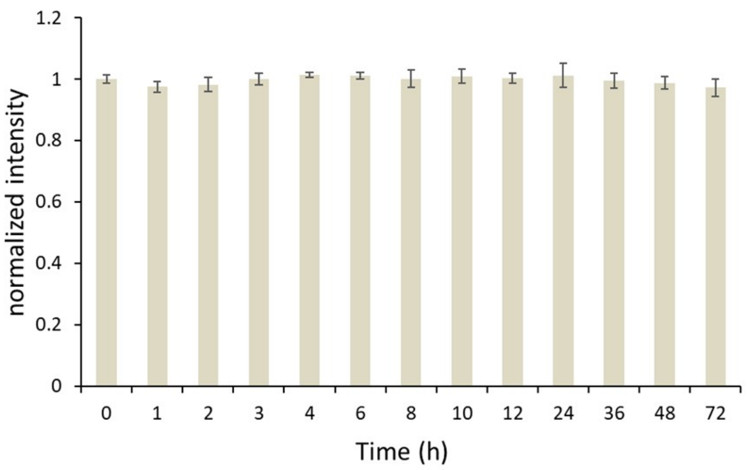

Fig. S5 The stability of TAPA. A series of 10 nM TAPA solution was prepared for the investigation of stability. 3 samples each time point, those samples were analyzed in a certain time from 0 – 72 h. In particularly, the intensity of TAPA at 72 h was 97.3% of the original intensity. Those results proved that TAPA could keep stable for at least 72 h.

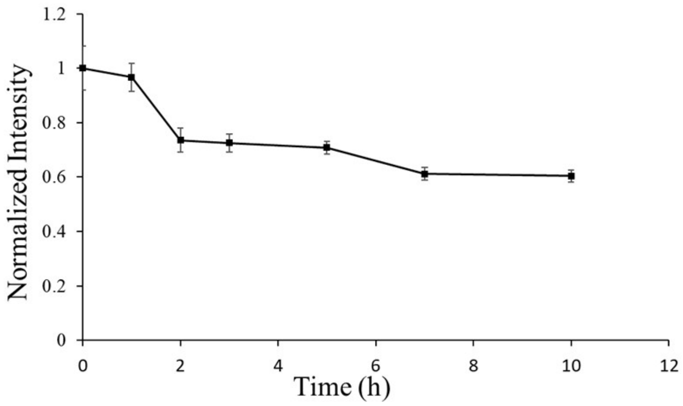

Fig. S6 The stability of TAPA in pH 3 solution. TAPA was still 60% left after 10 h through the addition of acid.

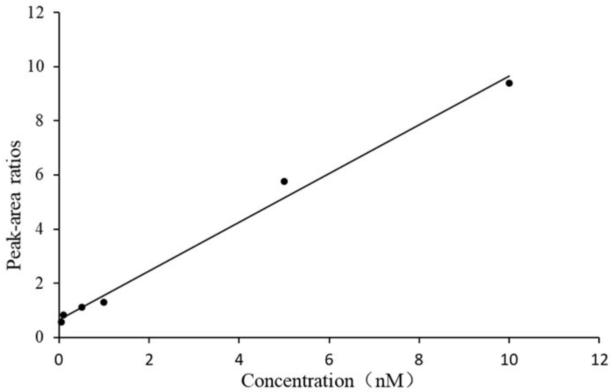

Fig. S7 Calibration curve: peak-area ratios versus the concentrations of thrombin (0.05 – 10 nM), the regression equation was y = 0.9002x + 0.6597 with a correlation coefficient of 0.9914.
